# *Indy* Mutations and *Drosophila* Longevity

**DOI:** 10.3389/fgene.2013.00047

**Published:** 2013-04-08

**Authors:** Blanka Rogina, Stephen L. Helfand

**Affiliations:** ^1^Department of Genetics and Developmental Biology, School of Medicine, University of Connecticut Health CenterFarmington, CT, USA; ^2^Department of Molecular Biology, Cell Biology and Biochemistry, Division of Biology and Medicine, Brown UniversityProvidence, RI, USA

**Keywords:** *Indy*, *Drosophila melanogaster*, aging and longevity, fruit flies, single gene mutation

## Abstract

Decreased expression of the fly and worm *Indy* genes extends longevity. The fly *Indy* gene and its mammalian homolog are transporters of Krebs cycle intermediates, with the highest rate of uptake for citrate. Cytosolic citrate has a role in energy regulation by affecting fatty acid synthesis and glycolysis. Fly, worm, and mice Indy gene homologs are predominantly expressed in places important for intermediary metabolism. Consequently, decreased expression of *Indy* in fly and worm, and the removal of *mIndy* in mice exhibit changes associated with calorie restriction, such as decreased levels of lipids, changes in carbohydrate metabolism and increased mitochondrial biogenesis. Here we report that several *Indy* alleles in a diverse array of genetic backgrounds confer increased longevity.

## Introduction

Aging is a complex process that can be modulated by environment and affected by genetic manipulations, such as single gene mutations. Understanding the underlying mechanisms by which single gene mutations extend life span can contribute to our understanding of the process of aging, and allow us to design therapeutic interventions that could postpone age-related decline and extend healthy aging. For example, based on the genetic data shown that down-regulation of the TOR signaling pathway extends longevity of yeast, worms, and fruit flies, experiments were performed that show that rapamycin, a drug that down-regulates the TOR signaling pathway, extends mice and fruit flies longevity (Vellai et al., 2003; Jia et al., 2004; Kapahi et al., 2004; Kaeberlein et al., 2005; Harrison et al., 2009; Bjedov et al., 2010).

Mutations in the *Indy* (*I’m Not Dead Yet*) gene extend life span of the fruit fly, *Drosophila melanogaster* (Rogina et al., 2000; Wang et al., 2009). Similarly, decreased expression of two of the worm *Indy* homologs extend worm longevity (Fei et al., 2003, 2004). *Indy* encodes the fly homolog of a mammalian di and tricarboxylate transporter involved in reabsorbing Krebs cycle intermediates, such as citrate, pyruvate, and α-ketoglutarate (Knauf et al., 2002, 2006; Pajor, 2006). Functional characterization of the transporter encoded by the *Indy* structural gene confirmed that it is a transporter of Krebs cycle intermediates (Inoue et al., 2002; Knauf et al., 2002). Studies in frog oocytes and mammalian cells showed that INDY mediates Na^+^, K^+^, and Cl^−^ independent high-affinity flux of dicarboxylates and citrate across the plasma membrane (Inoue et al., 2002; Knauf et al., 2002). Further studies have shown that INDY functions as an anion exchanger of dicarboxylate and tricarboxylate Krebs cycle intermediates (Knauf et al., 2006). Crystal structure of a bacterial INDY homolog from *Vibrio cholera* (VcINDY) reveals that one citrate and one sodium molecule is bound per protein but the mature transporter is likely found in the form of a dimer (Mancusso et al., 2012).

The fly INDY is most highly expressed in the gut, fat bodies, and oenocytes, all places where intermediary metabolism takes place, suggesting its role in metabolism (Knauf et al., 2002). Similarly, worm homologs (ceNaDC1 and ceNaDC2) are expressed in the intestinal tract (Fei et al., 2003), and the mouse gene *mIndy* (mINDY; SLC13A5) is predominantly expressed in liver (Birkenfeld et al., 2011). Based on INDY expression and a role in transporting Krebs cycle intermediates it has been hypothesized that decreased INDY activity creates a state similar to calorie restriction (CR). Studies in flies and mice support this hypothesis mainly by showing similarities between the physiology of *Indy* mutant flies and *mIndy* knockout mice on high calorie food and control flies and mice on CR (Wang et al., 2009; Birkenfeld et al., 2011).

It has recently been reported that longevity was not extended in worms with decreased levels of the *Indy* or in fruit flies with one of the alleles utilized by Rogina et al. (2000) and Toivonen et al. (2007). Toivonen et al. (2007), attributed the life span extension in *Indy* to the genetic background and bacterial infection (Toivonen et al., 2007). Subsequently, it was confirmed that the original *Indy^206^* mutation extends longevity after backcrossing into the *yw* background but not after backcrossing into the *w^1118^* genetic background (Wang et al., 2009; reviewed in Frankel and Rogina, 2012). Furthermore, it was demonstrated that the results published in Toivonen et al., are most likely due to differences in the caloric content of the food (Toivonen et al., 2007; Wang et al., 2009).

Here we report that the presence of one copy of an *Indy^206^* mutant chromosome extends longevity in several genetic backgrounds when compared to genetically matched controls. In order to further address the issues of *Wolbachia* contamination we treated the previously reported *Indy^159^* allele, and several new alleles, with tetracycline and backcrossed all of these *Indy* alleles into a *yw* genetic background for 10 generations. We determined survivorships of all *Indy* alleles on standard laboratory diet and found that several new *Indy* mutant alleles can also extend the longevity of male and female *Drosophila*. The data presented here further confirm the role of the *Indy* gene in *Drosophila* longevity and show the relationship between life span extension and reduction in *Indy* mRNA.

## Results

### Mutation in *Indy^206^* extends life span in different genetic backgrounds

**Figure 1:**
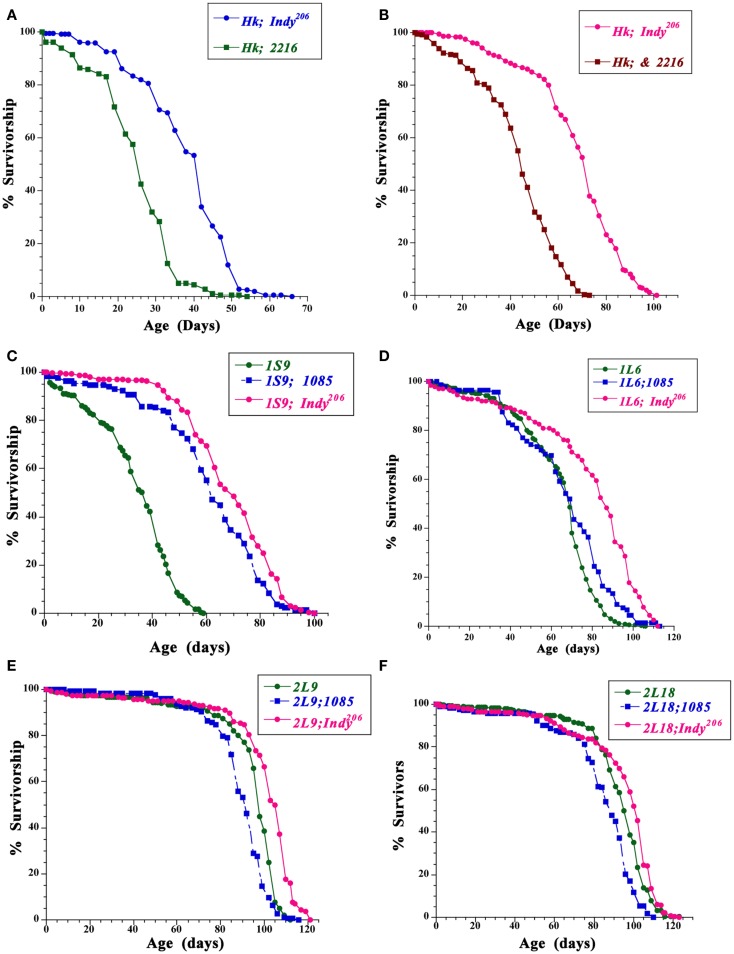
**Mutation in the *Indy* gene extends the life span of male and female *Drosophila* in different genetic backgrounds**. **(A,B)** Survivorships for male **(A)** and female **(B)** heterozygous for the *Indy^206^* (*Hk;Indy^206^*), and control (*Hk;2216*) enhancer-trap line in *Hyperkinetic* background. **(C–F)** Survivorship for male homozygous flies for the Luckinbill short live line 1S9 **(C)**, the Luckinbill long-lived line *1L6* **(D)**, *2L9* **(E)**, or *2L18* **(F)** and heterozygous for the *Indy^206^*, or 1085 and the Luckinbill *1S9*, *1L6*, *2L9*, or *2L18* lines at 25°C. Between 135–537 flies were used for each life span.

In order to further examine if genetic background may contribute to the life span extension of heterozygous *Indy* mutant flies, we determined the survivorship of *Indy* heterozygous mutant flies in *Hyperkinetic^1^ (Hk^1^*) and long- and short-lived selected Luckinbill lines (Figures 1A-E) (Kaplan and Trout, 1969; Luckinbill and Clare, 1985). *Hk^1^* is a recessive mutation characterized by hyperactivity and shorter life span of *Drosophila*. Hyperactivity is due to mutation of the beta (*Hk^1^*) subunit of the potassium channel, which causes increased neuronal excitability (Trout and Kaplan, 1970). *Hk^1^* is an X-linked recessive mutation, thus only male flies in those background live shorter (Trout and Kaplan, 1970; Rogina and Helfand, 1995). We used the *Hk^1^* line since it was isolated by an EMS mutagenesis of *Canton-S* (*CS*) stock in 1969 and therefore had many years of divergence from the *CS* background of the original *Indy* lines. We determined the survivorship of flies heterozygous for *Hk^1^* and either *Indy^206^* or control-*2216*. The 2216 and 1085 lines that were derived from the same mutagenesis as *Indy^206^*, but do not have a P-element insertions in the *Indy* region were used as control in Rogina et al., 2000. Survivorship analysis revealed that the median life span of male flies with one copy of the *Indy* mutation in *Hk* background is 52.0% increased as compared to the control *Hk;2216*. A similar increase in survivorship of 57.0% was observed in *Hk;Indy^206^* female flies when compared to the control females, Figures 1A,B; Table 1. (Median life span: *Hk;Indy^206^* males = 38.0 days, females = 68.0; *Hk;2216* males = 25.0, females = 43.3).

**Table 1:** **Life span of *Indy^206^* heterozygous flies is longer compared to the control flies in different genetic backgrounds**.

Gender	Genotype	*N*	Median life span (% change)	*X*^2^	*p*	Maximal life span (% change)
M	*Hk;Indy^206^/*+	210	38.0 (52.0)	146	*p* < 0.0001	53.6 (35.7)
M	*Hk;2216*	184	25.0			39.5
F	*Hk;Indy^206^/*+	294	68.0 (57.0)	286	*p* < 0.0001	91.0 (41.9)
F	*Hk;2216*	344	43.3			64.2
M	*1S9*	301	33.9			51.7
M	*1S9-Indy^206^*	284	67.4 (98.8)	526	*p* < 0.0001	89.5 (73.1)
M	*1S9-1085*	170	60.0 (77.0)	278	*p* < 0.0001	86.2 (66.7)
F	*1S9*	360	32.0			52.2
F	*1S9-Indy^206^*	245	52.6 (64.4)	252	*p* < 0.0001	83.5 (60.0)
F	*1S9-1085*	255	54.2 (69.5)	210	*p* < 0.0001	88.7 (69.9)
M	*1L6*	323	65.3			90.7
M	*1L6-Indy^206^*	240	78.8 (20.7)	169	*p* = 0	108.1 (19.2)
M	*1L69-1085*	135	66.9 (2.5)	7	*p* = 0.008	98.2 (8.3)
F	*1L6*	317	61.4			87.6
F	*1L6-Indy^206^*	258	75.4 (22.7)	155	*p* = 0	101.4 (15.8)
F	*1L69-1085*	154	48.7 (−20.7)	6.3	*p* = 0.01	90.5 (3.3)
M	*2L9*	291	93.0			106.3
M	*2L9-Indy^206^*	158	99.7 (7.2)	81.6	*p* < 0.0001	115.8 (8.9)
M	*2L9-1085*	177	88.9 (−4.4)	42.2	*p* = 8.3e−11	105.1 (−1.0)
F	*2L9*	340	80.0			99.6
F	*2L9-Indy^206^*	236	77.4 (−3.2)	17.9	*p* = 2.28e−05	104.5 (4.9)
F	*2L9-1085*	228	62.9 (−21.4)	140	*p* < 0.0001	87.9 (−12.0)
M	*2L18*	312	93.0			112.1
M	*2L18-Indy^206^*	457	94.1 (1.2)	21.7	*p* = 3.15e−6	114.1 (1.8)
M	*2L18-1085*	169	84.2 (−9.5)	42.9	*p* = 5.8e−1	105.3 (−6.0)
F	*2L18*	286	81.0			99.4
F	*2L18-Indy^206^*	537	73.2 (−9.6)	0.1	*p* = 0.764	100.7 (1.3)
F	*2L18-1085*	199	64.6 (−20.2)	112	*p* < 0.0001	85.6 (−14.0)

*M, males; F, females; *N*, number of flies in the experiment, *Hk*, *Hyperkinetic*; *1S9*, Luckinbill short *1S9* line; *1L6*, *2L9*, or *2L18* Luckinbill long-lived *1L6*, *2L9*, or *2L18* lines*.

### *Indy^206^* mutant heterozygous flies live longer in Luckinbill short- and long-lived lines compared to control lines

Luckinbill short- and long-lived lines were selected based on reproduction of a population of outbreed *Drosophila* early or late in life (Luckinbill and Clare, 1985). Selective breeding was carried out for 21 or 29 generations and resulted in a large difference in median longevity between short- and long-lived lines. For instance, median life span of males for the short-lived *1S9* line was 33.9 day, while median longevity for the long-lived line *1L6* = 65.3, *2L9* was 93.0 and *2L18* was 93.0 days. Similar differences in median longevity between short and long-lived line can be seen in females (Median longevity *1S9* = 32.0, *1L6* = 61.4, *2L9* = 80.0, and *2L18* = 81.0 days.) We examined if *Indy* mutant flies can affect longevity of Luckinbill short- and long-lived line differently as compared to controls and further extend the life of long-lived lines beyond that expected from hybrid vigor. Our data show that the *Indy^206^* mutation increases longevity of both short- and long-lived lines in all conditions, with one exception, the female *1S9;Indy^206^* flies have a similar median longevity compared to the controls. While, F1 heterozygous males flies from a cross between the control 1085 and the *1S9* short Luckinbill line show the expected life span extension due to hybrid vigor and have a 77% increase in median longevity as compared to the homozygous *1S9* line, F1 heterozygous *Indy^206^*;*1S9* male flies have much higher increase in median longevity of 98.8% compared to *1S9* homozygous flies, Figure 1C; Table 1. *Indy^206^* mutation further increased longevity of all long-lived Luckbill lines. F1 heterozygote animals from a cross between the *Indy^206^* enhancer-trap line and the laboratory selected long-lived line *1L6* of Luckinbill (*Indy^206^;1L6)* live 20.7% longer compared to the homozygous *1L6*. In contrast, heterozygous control *1085;1L6*, live only 2.5% longer then homozygous *1L6* flies. *2L9* homozygous long-lived Luckinbill line live much longer compared to *1L6* Figure 1D. However, *Indy* mutant heterozygous flies in *2L9* (*Indy^206^;2L9*) still live 7.2% longer compared to the *2L9* homozygous flies Figure 1E. In contrast, F1 heterozygous control males, *1085;2L9* have 4.4% shorter median life span compared to the homozygous *2L9* flies. Median male life span in days: *Indy^206^;2L9* males = 99.7, *1085;2L9* = 88.9, Figure 1E; Table 1. Thus, heterozygous *Indy^206^;2L9* male flies have an increase in life span of 12% over matched controls (*2L9;1085*), and 7.2% over the homozygote Luckinbill long-lived *2L9* line itself (Table 1). Median life span of female *1085;2L9* is decreased by 21.4% compared to median longevity of homozygous *2L9* female flies, while longevity of *Indy^206^;2L9* females is only 3.2% shorter compared to homozygous flies. (Median female longevity in days: *1085;2L9* = 62.9, *Indy^206^*;*2L9* = 77.4, Table 1). Heterozygous *Indy^206^* flies in the background of the *2L18* long-lived line do not live significantly longer compared to homozygous *2L18* flies; however, they live significantly longer compared to control *1085;2L18* heterozygous male flies, which live 9.5% shorter compared to *2L18* homozygous male flies. (Median male life span in days: *2L18* = 93.0, *Indy^206^*;*2L18* = 94.1, *1085;2L18* = 84.2, Figure 1F; Table 1). Similarly, *Indy^206^;2L18* heterozygous females live longer in *2L18* background compared to the controls.

### Life span extensions in different *Indy* alleles

**Figure 2:**
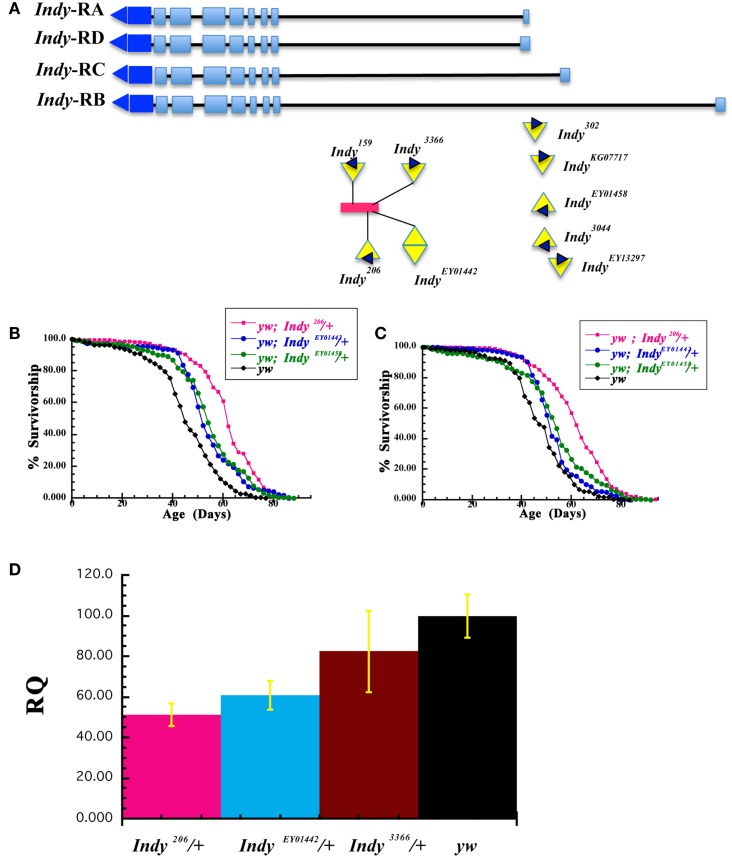
**Survivorships of different *Indy* mutant alleles in *yw* background**. **(A)** Genomic organization of the *Indy* locus with insertion sites and orientation of P-element in *Indy^206^*, *Indy^159^*, *Indy^EY01442^*, *Indy^3366^, Indy^302^*, *Indy^KG07717^, Indy^EY01458^*, *Indy^EP3044^*, and *Indy^EY013297^* alleles used in this manuscript. Orientation of P-element in *Indy^EY01442^*allele is not known. The red rectangle represents the conserved Hoppel transposable element. *Indy* encodes four putative transcripts (RA, RB, RD, and RC), which have different 5′ exon. **(B,C)** Life span of males **(B)** and females **(C)** heterozygous for *Indy^206^, Indy^EY01442^*, *Indy^EY01458^*, and *yw* on standard laboratory corn diet after 10× backcrossing into the *yw*. **(D)** *Indy* mutants have decreased levels of *Indy* mRNA. Q-PCR determination of *Indy* mRNA expression levels in Heads and Thorax of *Indy^206^*/+, *Indy^EY01442^*/+, *Indy^33662^*/+, and *yw* 20 days old male flies. Experiments were done in two (*Indy^206^*/+) or three (*Indy^EY01442^*/+, *Indy^3366^*/+, and *yw)* replicates with 2 × 15(*Indy^206^*/+), 3 × 40 (*Indy^3366^*/+), or 3 × 50 (*Indy^EY01442^*/+ and *yw)* flies in each group.

We have previously reported that five independent *Indy* mutant alleles extend the life span of male and female *Drosophila* in wild type *CS* and *yw* genetic backgrounds (Rogina et al., 2000; Wang et al., 2009). We have now tested an additional six *Indy* alleles for their effect on fly longevity (*Indy^EP3044^*, *Indy^EP3366^*, *Indy^EY01442^*, *Indy^EY01458^*, *Indy^EY013297^*, *Indy^KG07717^*). Genomic organization of the *Indy* locus and position of P-elements insertion in different *Indy* mutant alleles used in this manuscript is shown in Figure 2A. These six new alleles and three previously tested *Indy* alleles (*Indy^206^, Indy^302^, Indy^159^*) and *yw* control flies, were all treated with tetracycline to eliminate any possible bacterial contamination by *Wolbachia*. Although the absence of *Wolbachia* contamination after tetracycline treatment was not confirmed by PCR, we have previously confirmed the absence of Wolbachia after identical treatment (Wang et al., 2009). All of the *Indy* alleles and one of the control stocks 1085, which has the same genetic background as *Indy^206^*and *Indy^302^*, were backcrossed into the *yw* genetic background for 10 generations. We have determined longevity of all *Indy* alleles as heterozygotes in *yw* background and calculated median longevity for males and females, Table 2. Representative survivorships of two new *Indy* alleles are plotted in Figures 2B,C. Heterozygous *yw;Indy^206^/*+*, yw;Indy^302^/*+*, yw;Indy^159^/*+*, yw;Indy^EY01442^/*+ *yw;Indy^EY01458^/*+, and *yw;Indy^EY013297^*/+ male and female flies have a significantly longer life compared to control *yw* flies. Longevity extension in males with one copy of *Indy* mutant allele varies from 34.4 to 14.0%, and in females *Indy* mutant extension range from 29.4 to 10.7%, Table 2. In addition, female, but not male heterozygous *yw;Indy^EP3044^* flies live 9.2% longer compared to the controls. No effect on longevity was observed in male and female heterozygous *yw;Indy^KG07717^*/+, *yw;Indy^3366^*/+, and male heterozygous *yw;Indy^3044^*/+ mutant flies. We determined the levels of *Indy* mRNA isolated from Head& Thorax of male heterozygous for two of the new *Indy* alleles (*yw;Indy^EY01442^*/+, *yw; Indy^EP3366^/*+), one old (*yw;Indy^206^*/+), and their genetic control (*yw)*. The levels of *Indy* mRNA in heterozygous *yw;Indy^206^*/+ allele are 51.1% and in heterozygous *yw;Indy^EY01442^*/+ allele are 60.6% of the levels of *Indy* mRNA found in *yw* flies, Figure 2D. A similar decrease in the levels of *Indy* mRNA in *yw;Indy^206^*/+ was previously reported (Wang et al., 2009). We found only a minor, non-significant decrease in the levels of *Indy* mRNA in heterozygous *yw;Indy^EP3366^/*+ mutant flies. Lack of longevity effect in *yw;Indy^EP3366^/*+ allele is most likely due to only a small effect of the P-element insertion on the *Indy* mRNA levels in *yw;Indy^EP3366^/*+mutant flies. Our data show a strong correlation between the level of *Indy* mRNA and longevity extension.

**Table 2:** **Life span of several different *Indy* mutant alleles as heterozygous is longer compared to the control flies in *yw* genetic background**.

Gender	Genotype	*N*	Median life span (% change to *yw*)	*X*^2^	*p*	Maximal life span (% change)
M	*yw;Indy^206^*	224	61.3 (34.4)	146	*p* < 0.0001	78.9 (19.7)
M	*yw;Indy^302^*	161	52.0 (14.0)	14.1	*p* = 0.000164	69.0 (4.7)
M	*yw;Indy^159^*	169	54.5 (19.5)	35.8	*p* = 2.16e−09	69.2 (5.0)
M	*yw;Indy^EY01442^*	179	53.5 (17.3)	30.5	*p* = 3.37e−08	76.8 (16.7)
M	*yw;Indy^EY01458^*	151	53.8 (18.0)	33.5	*p* = 6.95e−09	75.5 (14.6)
M	*yw;Indy^EY13297^*	178	53.8 (18.0)	31.4	*p* = 2.15e−08	73.0 (10.8)
M	*yw;Indy^KG07717^*	178	45.9 (0.6)	0.9	*p* = 0.339	62.5 (−5.0)
M	*yw;Indy^EP3044^*	168	48.5 (6.3)	1.6	*p* = 0.207	75.3 (14.3)
M	*yw;Indy^EP3366^*	181	42.8 (−6.0)	12.1	*p* = 0.000499	57.2 (−13.0)
M	*yw;1085*	175	43.6 (−4.0)	5.5	*p* = 0.0191	62.3 (−5.0)
M	*yw*	169	45.6			65.9
F	*yw;Indy^206^*	316	61.7 (29.3)	149	*p* < 0.0001	80.7 (21.9)
F	*yw;Indy^302^*	192	52.8 (10.7)	10.8	*p* = 0.00102	71.9 (8.7)
F	*yw;Indy^159^*	212	56.5 (18.4)	51.5	*p* = 6.15e−13	76.9 (16.3)
F	*yw;Indy^EY01442^*	186	52.8 (10.7)	11.4	*p* = 0.000731	74.1 (12.0)
F	*yw;Indy^EY01458^*	206	53.0 (11.1)	24.5	*p* = 8.97e−07	79.4 (20.0)
F	*yw;Indy^EY13297^*	190	54.7 (14.7)	30.5	*p* = 3.36e−08	76.1 (15.0)
F	*yw;Indy^KG07717^*	181	48.6 (1.9)	0.1	*p* = 0.795	70.3 (6.3)
F	*yw;Indy^EP3044^*	186	52.1 (9.2)	7.8	*p* = 0.00522	76.9 (16.2)
F	*yw;Indy^EP3366^*	189	48.9 (2.5)	2.4	*p* = 0.119	57.3 (−13.0)
F	*yw;1085*	187	48.4 (1.4)	2.2	*p* = 0.141	59.2 (−11.0)
F	*yw*	200	47.7			66.2

*M, males; F, females; *N*, number of flies in the experiment*.

## Discussion

We have previously identified and characterized five independent mutations in the *Indy* gene in *Drosophila* that cause an increase in average and maximal life span for both male and female fruit flies (Rogina et al., 2000). The original five alleles were derived from three different mutageneses (Boynton and Tully, 1992; Rogina et al., 2000). Life spans of flies carrying one copy of P-element in the *Indy* gene were compared with their close genetically matched controls, flies from the same mutagenesis without a P-element insertion in the *Indy* gene. Here we show that *Indy^206^* heterozygous mutant flies also live longer when crossed into three different genetic backgrounds, *Hk*, short, and long-lived Luckinbill lines as compared to control flies from the same genetic background as *Indy* also crossed to these three different genetic backgrounds. Luckinbill short and long-lived lines have been generated by selective breeding for early and late female fecundity (Luckinbill and Clare, 1985). Presence of the *yw;Indy^206^* mutant chromosome significantly extends longevity in the background of the Luckinbill short *1S9* line compared to the control line 1085. Moreover, the *Indy^206^* mutation further extends longevity of two long-lived Luckinbill lines and does not cause shortening of life span of *2L1*8 long-lived line. At the same time, median longevity of control lines when crossed to Luckinbill long-lived lines are significantly shorter compared to homozygous Luckinbill lines. These data show that extension of life span by this *Indy* allele is not limited to the background of the short-lived lines, but further extends lines already selected for long life span.

We also report extension of longevity by additional *Indy* mutant alleles. All *Indy* mutant alleles were treated by tetracycline to prevent any effects of *Wolbachia* and backcrossed to *yw* background. *Wolbachia* infection was proposed as a contributing factor to *Indy* longevity by Toivonen et al. (2007). *Indy^EY01442^*, *Indy^EY01458^*, *Indy^EY013297^*, *Indy^KG07717^* were generated by the Berkeley *Drosophila* Genome Project (BDGP) gene disruption project (Bellen et al., 2004). The *Indy* gene region appears to be a “hot spot” for P-element insertions illustrated by isolation of 5 KG, 28 EY, and 10 EP element insertions in the *Indy* region (Bellen et al., 2004). P-element insertion in *Indy^206^*, *Indy^159^*, *Indy^EY01442^*, and *Indy^EP3366^* are within the Hoppel element in the first intron of the *Indy* gene, upstream of the putative translational start site, Figure 2A. The conserved Hoppel element is present in the same position in wild type flies (Rogina et al., 2000). The insertion in *Indy^302^*, *Indy^EY013297^*, *Indy^EY01458^*, *Indy^KG07717^*, and *Indy^EP3044^* lines is upstream from putative transcriptional start sites. *Indy* encodes four putative transcripts, which have different 5′-exons. The positions of P-elements in *Indy^302^*, *Indy^EP3044^*, *Indy^EY01458^*, *Indy^EY013297^*, and *Indy^KG07717^* are located close to the three putative transcriptional start sites for three putative *Indy* transcripts (*Indy-RA, Indy-RD*, and *Indy-RC*) and about 5,000 bp upstream from the putative transcriptional start site in *Indy-RB*. Genomic organization of the *Indy* locus and positions of P-element insertion in different *Indy* alleles used in this manuscript are shown in Figure 2A. Positions of additional P-elements insertion can be seen in FlyBase: http://flybase.org/reports/FBgn0036816.html. It was previously shown that the presence of the P-element in *Indy^206^* and *Indy^302^* mutant alleles decreases the levels of *Indy* mRNA most likely by affecting transcription (Knauf et al., 2006; Wang et al., 2009). The levels of *Indy* mRNA are decreased about 95% in homozygous *Indy^206^* and about 40% in homozygous *Indy^302^* alleles (Wang et al., 2009). The levels of INDY protein are also dramatically decreased in *Indy^206^* homozygous mutant flies (Knauf et al., 2002). Similarly, here we show that the levels of *Indy* mRNA are decreased about 39% in the heterozygous *Indy^EY01442^*/+ allele and about 49% in the heterozygous *Indy^206^*/+ allele compared to the levels of *Indy* mRNA found in *yw* flies. No significant decrease in the levels of *Indy* mRNA were observed in heterozygous *Indy^3366^*/+ flies, which correlates with the absence of longevity extension. It is likely that variation in longevity effects of different *Indy* alleles correlates to actual *Indy* mRNA levels and differential effects of P-elements on transcription. We found that male flies heterozygous for six *Indy* alleles have longevity extension ranging from 14.0 to 34.4%. Females heterozygous for seven *Indy* alleles show similar result having longevity extension ranging from 9.2 to 29.3%. Our data further confirm our hypothesis that the level of *Indy* expression is central for longevity extension. When the levels of *Indy* mRNA are decreased approximately 49%, as in *Indy^206^*/+ heterozygous mutant flies, there is dramatic longevity extension of 34%. We have previously reported that when the levels of *Indy* mRNA are radically reduced, as in *Indy^206^* homozygous flies, longevity extension is less than extension of the *Indy^206^*/+ heterozygous flies (Wang et al., 2009). A smaller longevity effect of 17% was observed when *Indy* mRNA levels are moderately reduced, as in *Indy^EY01442^*/+. Insignificant reduction of *Indy* mRNA levels, as in *Indy^EP3366^*/+ mutant flies, resulted in no longevity effect. Besides *Indy^EP3366^*/+, no longevity extension was found in another one of the new alleles, *Indy^KG07717^*. In summary, maximal longevity in *Indy* mutant flies is associated with optimal reduction of *Indy* mRNA levels. When *Indy* levels are too low or close to normal, longevity effects are diminished. Although a recent report attributed life span extension in *Indy* to hybrid vigor, due to life span evaluation in an incorrect genetic background, and bacterial infection, our data presented here corroborate a link between the *Indy* mutations and longevity in flies (Toivonen et al., 2007; Wang et al., 2009). The effect of the *Indy* mutation on longevity was supported by findings that decreased activity of NaDC2, a *C. elegans* homolog of the *Indy* gene, extends the life span of worms (Fei et al., 2003, 2004). Similar effects of increased longevity associated with mutations in the fly and the worm *Indy* gene suggests a possibility of evolutionary conservation and a universal role of INDY in longevity (Fei et al., 2003, 2004).

Several studies have investigated the molecular mechanisms underlying the effects of the *Indy* mutation on longevity and health span of worms, flies, and mice (Fei et al., 2003; Marden et al., 2003; Neretti et al., 2009; Wang et al., 2009; Birkenfeld et al., 2011). INDY is a plasma membrane transporter that may mediate the movement of dicarboxylic acids through the epithelium of the gut and into organs important in intermediary metabolism and storage (Knauf et al., 2002, 2006). Location of the INDY transporter in the fat body and oenocytes suggest a role in intermediary metabolism and expression in the gut suggests a role in uptake of nutrients. Reductions in INDY activity may alter uptake, utilization, or storage of important nutrients and affect normal metabolism. It has been hypothesized that reductions in *Indy* activity seen in *Indy* mutations might be altering the normal energy supply in flies resulting in life span extension through a mechanism similar to CR. CR has been shown to increase life span and delay the onset of age-related symptoms in a broad range of organisms (McCay et al., 1935; Weindruch and Walford, 1988). Consistent with the hypothesis that *Indy* is important in metabolism is the finding that *Indy* mutant worms, flies, and mice have disrupted lipid metabolism (Fei et al., 2003; Wang et al., 2009; Birkenfeld et al., 2011). Similarly to CR animals, *Indy* mutant flies have increased spontaneous physical activity, decreased starvation resistance, weight, egg production, and insulin signaling. Furthermore, wild type flies on CR have significantly decreased levels of *Indy* mRNA (Wang et al., 2009). *Indy* homozygous mutant flies live shorter on low calorie foods compared to controls, which is consistent with our hypothesis that *Indy* mutant flies are already in a state of reduced nutrition on normal food and when food is further reduced, life span is shortened due to starvation (Wang et al., 2009). In addition, *Indy* mutant flies have increased mitochondrial biogenesis in heads and thoraces similar to CR animals (Neretti et al., 2009). Similarly, *mIndy* knockout mice have increased mitochondrial biogenesis in the liver. The mechanism of the effect of a decrease in INDY on metabolism is likely from its physiological function as a citrate transporter. Cytosolic citrate is the main precursor for the synthesis of fatty acid, cholesterol, triacylglycerols, and low-density lipoproteins. In addition, cytosolic citrate inhibits glycolysis and fatty acid β-oxidation. Therefore, INDY by affecting the levels of cytosolic citrate may alter glucose and lipid metabolism in a manner that favors longevity. Additional support that *Indy* mutation mimics CR comes from the findings that *mIndy* knockout mice are protected against adiposity and insulin resistance when kept on high fat diet (Birkenfeld et al., 2011). The data from worm, fly, and mice studies highlight the importance of INDY in health span and longevity. New *Indy* alleles described here should provide additional tools to further explore the role of INDY in metabolism and its connection to extended longevity and health.

## Materials and Methods

### Fly strains

*1S9* a short-lived and *1L6, 2L9*, and *2L18* long-lived lines were a kind gift from James W. Curtsinger and originally described in Luckinbill and Clare (1985). *Indy^206^*, *Indy^302^*, 1085, and 2216 were obtained from Tim Tully (Boynton and Tully, 1992). *Indy^159^* was kind gift from the Bier lab (Bier et al., 1989). *Indy^EP3044^*, *Indy^EP3366^*, *Indy^EY01442^*, *Indy^EY01458^*, *Indy^EY013297^*, *Indy^KG07717^*alleles, and *Hk^1^* were obtained from the Bloomington Stock Center or Exelexis. Heterozygous flies used in survivorship analysis are F1 generations from crosses in which virgin females homozygous for *Hk^1^*, short-lived, long-lived Luckinbill lines, or *yw* were mated to males homozygous for different *Indy* alleles, or the control lines 1085 or 2216.

### Backcrossing scheme

*Indy^206^, Indy^302^, Indy^159^, Indy^EP3044^*, *Indy^EP3366^*, *Indy^EY01442^*, *Indy^EY01458^*, *Indy^EY013297^*, *Indy^KG07717^*, and 1085 were backcrossed into the *yw* background. Female virgins from *yw* were mated with males of different *Indy* alleles or *1085*. Heterozygous females were then backcrossed to *yw* males for 10 generations.

### Food recipe

We used standard yeast, corn, sucrose food in our experiments: 113 g Sucrose (MP Biomedicals, Fischer Scientific) and 28 g Brewers yeast (MP Biomedicals, Fischer Scientific) was mixed with 643 ml water and autoclaved for 20 min. 49 g corn (MP Biomedicals, Fischer Scientific) and 8.1 g Agar (SciMart) were mixed in 268 ml water and added to the food mixture and autoclaved for 20 min. The food was cooled down with constant mixing. 2.4 g tegosept (Fischer Scientific) dissolved in 10.7 ml 100% EtOH was added when the food temperature was 65°C. Approximately 10 ml food was poured to plastic vials using Fly food dispenser (Fischer Scientific), and vials were covered with Kimwipes and cheese cloth. Once the food was cooled down it was stored at 4°C. Before use the food was warmed up to room temperature.

### Life span

Vials were cleared of adult flies in the morning and the collection of newly eclosed flies occurred in the afternoon. Approximately 20 male and 20 female flies were kept together in a plastic vials with approximately 5–10 ml of a standard cornmeal media (Rogina et al., 2000). Flies were housed in humidity-controlled incubators, maintained at 25°C on a 12 h light: dark cycle. Vials of fresh food were supplied three times weekly (Monday, Wednesday, and Friday) and the number of dead flies was recorded during each passage from old to new vials.

### mRNA isolation Q-PCR analysis

The standard Chomczynski protocol and Trizol reagent (Gibco BRL) were used to isolate mRNA (Chomczynski and Sacchi, 1987). Male flies at age 20 were placed on a cold block and Head with Thorax were dissected. Three biological replicates of 50 males were used in each isolations of *Indy^EY01442^*/+ and *yw* flies, three biological replicates of 40 *Indy^3366^*/+ males and two biological replicates of 15 *Indy^206^*/+ males. Q-PCR was performed with *Indy* and Ankyrin specific primers obtained from Applied Biosystems according to the manufacturers protocol. Ankyrin was used as an endogenous control. The samples were run on the AB 7500 System.

### Statistical analysis

Life span data were analyzed by long-rank tests (http://bioinf.wehi.edu.au/software/russell/logrank/). Maximum life span was calculated as the median life span of the longest surviving 10% of the population.

## Conflict of Interest Statement

The authors declare that the research was conducted in the absence of any commercial or financial relationships that could be construed as a potential conflict of interest.
